# Ileal conduit post radical cystectomy: modifications of the technique

**DOI:** 10.3332/ecancer.2013.301

**Published:** 2013-04-04

**Authors:** Ahmed Fouad Kotb

**Affiliations:** FEBU Urology Department, Alexandria University, Al-Khartoum square, PO 12345, Alexandria, Egypt

**Keywords:** Bladder cancer, Radical cystectomy, Ileal conduit, Pelvic kidney

## Abstract

**Introduction:**

Ileal conduit, post radical cystectomy, is currently the most practiced type of urinary diversion. The aim of our study was to modify the ileal conduit technique in order to retain acceptable outcomes while decreasing the rate of postoperative urine leak and ureteroileal stricture.

**Methods:**

Forty consecutive patients were included in our study, from January to June 2011. Radical cystectomy and ileal conduit using our modifications were done for all the cases. Follow-up was done for one year. One patient with right pelvic kidney was added to the studied group and was managed by extra modification to our technique.

**Results:**

The mean age of the patients was 63 years. No significant leak and no stricture were observed within the modest duration of follow-up. When a left upper urinary tract retrograde study was attempted in one patient, the left ureteric orifice could not be reached due to a redundant elongated ileum.

**Conclusions:**

Modified Bricker techniques are safe, avoid early postoperative urine leak, and lower the incidence of ureteroileal anastomotic stricture. Using our modifications, retrograde access of the left ureter may not be possible. The ‘Z’ ileal conduit is a feasible technique that can allow tension-free healthy ureteroileal anastomosis for cases with pelvic right kidney and, probably, post renal transplantation.

## Introduction

Radical cystectomy represents the standard of care for the majority of men with muscle-invasive bladder cancer. The ileal conduit is currently the most adopted type of urinary diversion post radical cystectomy. Recently, Colombo *et al* [1] confirmed ileal conduits to be the appropriate surgical solution for most patients post radical cystectomy. The ileal conduit technique was popularised by Bricker [2] and depends on the use of a short bowel segment that is anastomosed to both ureters and transfers the urine to an incontinent stoma.

A big issue that urologists encounter during urinary diversion is left ureteroileal anastomosis. The surgeon is often concerned about resecting the distal segment of the ureter for proper oncological outcomes, while needing to have a long left ureter, to pass it through the sigmoid mesentery for anastomosis to the short ileal conduit on the right side. The rate of carcinoma *in situ* in the distal ureter at the time of radical cystectomy has been reported to be 8.5–33% of cases [3], adding a surgical burden for the resection of the distal ureter and following the result of the frozen section pathology.

Tension on the ureteroileal suture line would result in urine leak in the early postoperative period and would end in ureteric stricture on long-term follow-up [1, 4]. Kouba *et al* [5] have reported a higher rate of ureteroileal stricture in Bricker ileal conduits and attributed that to the high body mass index of their patients, so obese patients represent an extra surgical burden to the difficulties involved in tension-free left ureteroileal anastomosis.

Ectopic kidney represents a rare congenital anomaly that occurs in about 0.2% of the general population, with pelvic kidney representing nearly one-quarter of such cases [6, 7]. The incidence of end-stage renal disease is high, presenting approximately 0.4% of populations [8], and it is getting higher with increasing age. Renal transplantation is a curative solution for the majority of these cases. Few reports have been published discussing the ileal conduit diversion in association with renal transplantation.

The objective of our work was to modify the ileal conduit technique to keep acceptable outcomes while decreasing the rate of postoperative urine leak and ureteroileal stricture. A case report was included with the main research work to add a certain modification that may be of value for cases, with congenitally right pelvic kidney or post renal transplantation.

## Methods

We are a tertiary referral institution for management of uro-oncology cases. All men with invasive bladder cancer were counselled about different types of urinary diversion post radical cystectomy. Patients who chose ileal conduits were recruited to our study group. Forty consecutive patients were included in our study, from January to June 2011. Follow-up was done after one year for each patient in the studied group. All the studied cases were men referred to us with invasive bladder tumours and normal renal functions. In our institution, we prefer making women stoma free, and so we manage them either with the modified ureterosigmoidostomy technique or by doing continent reservoir.

The technique involves isolation of a long segment of ileum, 15cm from the ileocecal junction. The proximal end of the segment was passed through the sigmoid mesocolon to the left side and then sutured to the left psoas muscle for proper fixation. The shorter the left ureter, the longer the ileal segment we isolate, thus allowing the conduit to reach the left ureter, in its natural place, without excessive dissection.

**Figure 1:**
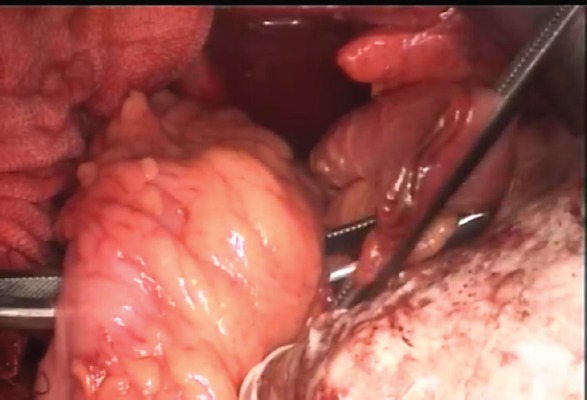
Getting the ileal conduit through the sigmoid mesocolon.

**Figure 2:**
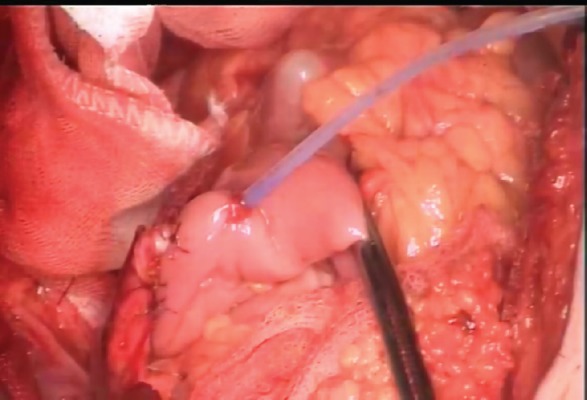
Left ureteric stent, coming out through an ileal puncture, close to the left ureteroileal anastomosis.

**Figure 3:**
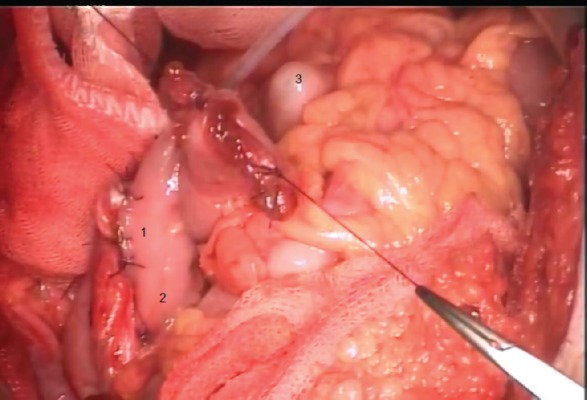
1. Full-thickness suture line; 2. proximal seromuscular fixating sutures; 3. rectal tube, within the sigmoid colon.

We spatulate the ureteric orifices, then we suture the ureteric serosa, 1 cm above the level of spatulation, to the ileal seromusculosa. We believe this allows more fixations for the anastomosis and avoids direct tension on the ureteroileal anastomosis. The ileum is then widely opened by a stab, and a watertight anastomosis is done over an 8-Fr nasogastric tube. The right nasogastric tube comes out through the ileal stoma. The left nasogastric tube comes out through a ureteric puncture on the left side of the conduit, close to the left ureteroileal anastomosis, and it is tightened by a purse-string suture. Figures 1–3 illustrate key steps in the surgical technique.

## The case with right pelvic kidney (case 41)

**Figure 4:**
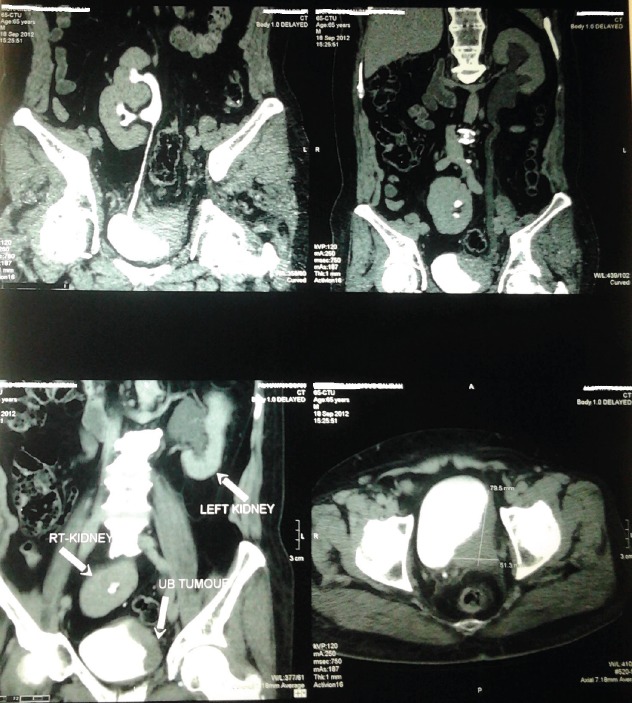
Preoperative CT findings: illustrations showing right pelvic kidney and a large urinary bladder tumour encroaching on the left ureteric orifice.

Our technique was applied for a 65-year-old man, who was presented to our institution with a muscle-invasive bladder tumour, which was infiltrating the distal part of the left ureter, and a right pelvic kidney. Figure 4 shows preoperative CT findings of the patient. Radical cystectomy and bilateral pelvic lymph node dissection were done. The distal part of the left ureter was excised until the frozen section pathology came back negative for malignancy.

**Figure 5:**
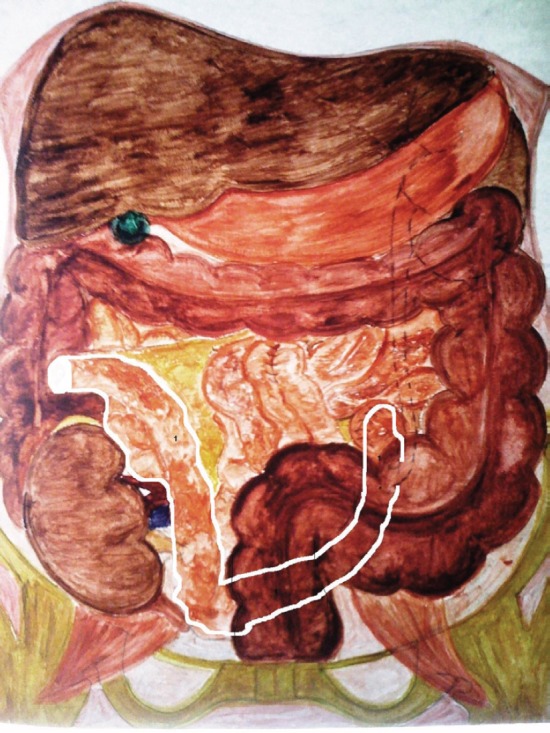
Diagrammatic illustration of the ileal conduit ‘Z’ technique. 1. ileal conduit; borders marked with white colour.

**Figure 6:**
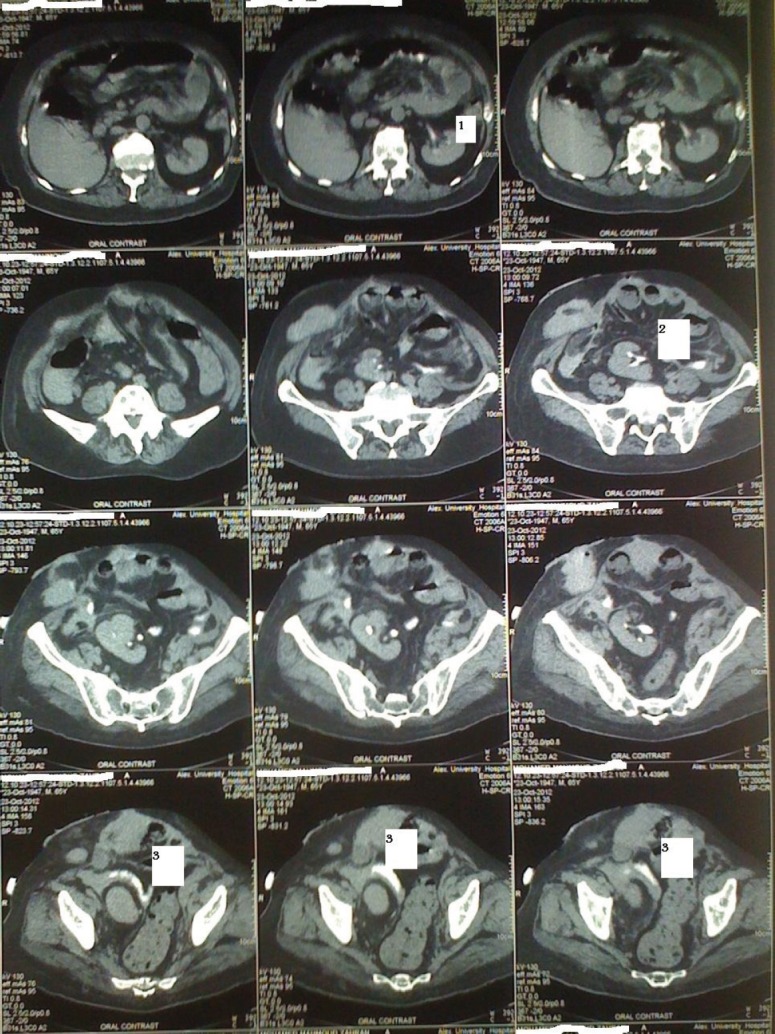
Figure 6: Postoperative CT findings. Dye is present in the conduit filling. The right and left pelvicalyceal systems: 1. Dye in the left kidney; 2. dye in the right kidney; 3. ‘Z’ conduit, passing above right kidney, and descending between the kidney and sigmoid colon.

The ileal conduit was constructed in a ‘Z’ shape so that the distal end forms the ileostomy. Then the ileal segment was passed distally on the medial aspect of the right pelvic kidney, extending upward and directed to the left side, below the sigmoid mesocolon. The right ureter could then be easily anastomosed to the ileal segment, just passing beside it. The seromuscular layer of that descending ileal limb was sutured to the seromuscular layer of the sigmoid colon. The ascending limb behind the sigmoid mesocolon was then fixed to the left psoas muscle and used for anastomosis, with the short left ureter. Figure 5 represents a diagrammatic description of the technique. CT loopogram was done, one month later, injecting dye through the ileostomy and showing the course of the conduit and the reflux of dye into collecting system of both kidneys. Figure 6 shows the loopogram.

## Results

The mean age of the patients was 63 years. All patients had muscle-invasive urothelial bladder tumours. All were followed for one year. All cases demonstrated asmooth early postoperative period. No urine leak was noticed during hospital stay. An oral diet was resumed on the third postoperative day, and drains were removed on the fifth day. Ureteric stents were removed two weeks after surgery.

At the one-year follow-up, 39 patients had smooth course, with no loin pain and no hydronephrosis. One patient had persistent left loin pain. A CT urogram was done, suggesting left pelviureteric junction obstruction, with the presence of a dilated, infected left kidney. Left percutaneous nephrostomy was inserted, and a descending nephrostogram was done, revealing a large filling defect in the renal pelvic region. A decision was made to do left flexible uretroscopy through the ileal stoma. The surprising finding was that the ileal segment became very redundant and elongated to the extent that the flexible uretroscope could not reach the left ureteroileal anastomosis.

The case presented with pelvic kidney had a smooth postoperative course, but wound infection was managed medically (Clavien grade II).

## Discussion

The ileal conduit is currently the most adopted mode of urinary diversion post radical cystectomy. We believe that to reach the desired good oncological outcome, the surgeon should try to excise all suspicious segments of the ureters without being worried about the next step, urinary diversion. Left ureteroileal anastomotic stricture is encountered in a significant number of cases. Possible explanations include ischaemia of the distal ureter due to excessive mobilisation and tension on the ureter caused by the crossing of the left ureter through the sigmoid mesocolon [9]. An advantage of our modifications was to do the reverse, crossing the proximal end of the ileal conduit through the mesocolon, to the left side, allowing tension-free ureteroileal anastomosis, which would result in a low rate of early urine leak and low rate of later anastomotic stricture. This modification also allowed lower need for mobilisation and dissection of the ureter, avoiding ischaemia as a possible cause of left ureteroileal stricture.

While following our cases, we could find other groups of enthusiastic urologists thinking in the same way that we did. Li *et al* [10] managed a series of 42 patients, post radical cystectomy, by the modified Bricker technique. They used a longer segment of ileum that was crossed to the left side through the sigmoid mesocolon. In our series, we used a long ileal segment that was crossed to the left ureter, through the sigmoid mesocolon, avoiding excessive dissection and mobilisation of the left ureter. To avoid tension on left ureteroileal anastomosis, resulting from ileal peristalsis, we added two more steps: the first is the fixation of the crossed conduit to the left psoas muscle, and the second is the 1-cm seromuscular suturing of the terminal ureter to the ileal wall, which would avoid direct tension on the anastomotic line.

Our series had a very smooth early postoperative period, with no evidence of urine leak and with early ambulation. At one-year follow-up, none of our patients had developed anastomotic stricture. However, when the decision was made to do a retrograde endoscopic manoeuver to the left ureter, it could not be done, due to excessive redundancy of the ileal conduit, making the left ureteric meatus far from endoscopically accessible, a condition that represented a limitation to our technique. In that case, percutaneous antegrade access was obtained.

Urinary diversion post radical cystectomy in the presence of a pelvic kidney or renal transplant may be very confusing to the urologist. Only a few cases were described in the literature. Surange *et al* [11] studied 54 patients of renal transplants that were drained into ileal conduits. They used the ordinary ileal conduit technique, and during transplantation, they inverted the kidney, so the ureter took a short non-kinked course to the conduit. Urine leak was a complication in the first two weeks of transplantation. Chaykovska *et al* [12] managed six cases, and they also transplanted the kidney upside down, but they did uretro-uretrostomy with the native ureter that was anastomosed to the ileal conduit. In previous cases, radical cystectomy was planned before renal transplantation, so transplanting the kidney upside down might be a suitable solution.

In our case, the kidney was congenitally pelvic in location, so we had to do a modification of the ileac conduit, allowing tension-free right ureteroileal anastomosis. Quek *et al* [13] recently published a case report of a similar case, for which they used a longer ileal conduit to allow easier ureteroileal anastomosis. In their case, the left ureter was long enough to cross the right side below the sigmoid mesocolon. Tomaszewski *et al* [14] reported three cases presenting with invasive bladder tumour following kidney transplantation: two of them underwent radiotherapy, plus ileal conduit diversion in one of them, with no comment on the technique applied. The third case was managed by radical cystectomy and allograft nephrostomy.

We believe our modifications to the traditional ileal conduit not only allow tension-free right ureteroileal anastomosis but also ensure a viable anastomotic anastomosis by avoiding extra dissection of the short right ureter. In our case, the ureter was not mobilised at all, and the ileum was pulled down to the ureteric location. Ileal fixation to the sigmoid by seromuscular sutures keeps the ileal segment in its modified position. The technique is easy to do and can be reproduced in cases with pelvic kidney and possibly following renal transplantation.

Our work has taught us that we are still not offering our patients the best standard of care. Our modifications to the traditional Bricker technique may result in decreasing the rate of early postoperative urine leakage and late ureteroileal stricture. On the other hand, a drawback of the modifications is that the left ureter may not be accessible for any possible future retrograde endoscopic intervention. Longer follow-up and more cases will be required to prove real long-term benefits. Hopefully, we can keep upgrading our techniques to provide the best possible care for patients with bladder cancer.

## Conclusion

Modified Bricker techniques are safe, reproducible, avoid early postoperative urine leak, and lower the incidence of ureteroileal anastomotic stricture. Using our modifications, retrograde access of the left ureter may not be possible. The modified ‘Z’ ileal conduit is a feasible technique that can allow tension-free healthy bilateral ureteroileal anastomosis for cases with right pelvic kidney and probably post renal transplantation.
